# Association of weight-adjusted waist index with preserved ratio impaired spirometry and all-cause mortality

**DOI:** 10.3389/fnut.2025.1594453

**Published:** 2025-05-20

**Authors:** Xin Jiao, Linqi Huang, Luoqi Lin, Fanggang Zhu, Feiting Fan, Jingmin Xiao, Lei Wu, Lin Lin, Yuanbin Chen

**Affiliations:** ^1^State Key Laboratory of Traditional Chinese Medicine Syndrome, The Second Clinical College of Guangzhou University of Chinese Medicine, Guangzhou, China; ^2^Guangdong Provincial Hospital of Chinese Medicine, The Second Affiliated Hospital of Guangzhou University of Chinese Medicine, Guangzhou, China; ^3^Guangdong-Hong Kong-Macau Joint Lab on Chinese Medicine and Immune Disease Research, Guangzhou University of Chinese Medicine, Guangzhou, China; ^4^The First Clinical Medical College of Guangzhou University of Chinese Medicine, Guangzhou, China

**Keywords:** preserved ratio impaired spirometry, weight-adjusted waist index, NHANES, mortality, obesity

## Abstract

**Background:**

Preserved ratio impaired spirometry (PRISm) is considered an early indicator of chronic obstructive pulmonary disease (COPD). This study aimed to investigate the association between weight-adjusted waist index (WWI) and PRISm, and the impact of WWI on all-cause mortality in U.S. adults with and without PRISm.

**Methods:**

This combined cross-sectional and cohort study analyzed data from 9,841 participants in the 2007–2012 National Health and Nutrition Examination Survey (NHANES). Weighted logistic regression assessed the association between WWI and PRISm. Kaplan–Meier survival curves and weighted Cox regression evaluated the effect of WWI on all-cause mortality. Restricted cubic spline (RCS) analysis explored both linear and nonlinear relationships between WWI and outcomes.

**Results:**

After covariate adjustment, each unit increase in WWI was associated with a 45% reduced risk of PRISm (ORs = 0.55; 95% CIs: 0.47–0.65). RCS analysis revealed a nonlinear WWI-PRISm relationship (*p* for nonlinearity = 0.0012). In the PRISm population, each WWI unit increase associated with an 88% higher adjusted all-cause mortality risk (HRs = 1.88; 95% CIs: 1.38–2.56). A U-shaped curve characterized the nonlinear WWI-mortality association in PRISm (*p* for nonlinearity = 0.0025), whereas positive linear trends were observed in non-PRISm individuals and overall.

**Conclusion:**

Lower WWI levels were linked to an elevated PRISm risk, highlighting central obesity’s role in respiratory health. Maintaining an optimal WWI may mitigate mortality risk in adults with PRISm, emphasizing the need for targeted weight management.

## 1 Introduction

Chronic obstructive pulmonary disease (COPD) is currently the third leading cause of mortality globally, causing 3.23 million deaths in 2019 and imposing a substantial burden on healthcare systems (1). Preserved ratio impaired spirometry (PRISm) is defined as a forced expiratory volume in 1 second (FEV_1_)/forced vital capacity (FVC) ratio ≥0.7 and FEV_1_ <80% of the predicted value, with a global prevalence reported between 7.1% to 20.3% (2–4). Compared with normal spirometry, those with PRISm exhibit higher morbidity and poor health outcomes, including increased respiratory symptoms, decreased exercise tolerance, and higher rates of respiratory-related hospitalizations and fatalities (2, 5, 6). PRISm does not always normalize over time, and 20–30% of individuals progress to obstructive ventilation dysfunction (7). Research has indicated that the PRISm population is more likely to be clinically diagnosed with asthma and COPD (2). The 2024 Global Initiative for Chronic Obstructive Lung Disease (GOLD) guidelines noted that the PRISm population should be regarded as “patients” and receive care and treatment (7). PRISm may be a precursor of COPD in some individuals, and its reversibility is particularly important (6, 8). Despite the increasing attention that PRISm has received, the mechanisms of PRISm development, longitudinal trajectories, and therapeutic management have not been fully described. Given the substantial effects that COPD has on population health and economy, early identification of PRISm and suitable interventions are crucial for COPD prevention.

Obesity is a significant risk factor for chronic noncommunicable diseases worldwide and is closely related to increased disease incidence and mortality (9). Several studies have demonstrated a strong correlation between obesity and inflammatory respiratory conditions, such as COPD and asthma (10–12). Previous research has identified risk factors for PRISm, including advanced age, smoking, and an abnormal body mass index (BMI) (13). Notably, both low and high BMI were associated with an increased risk for PRISm (13). Recently, central obesity has been recognized as a more representative manifestation of obesity-related systemic inflammation and metabolic damage (14). Central obesity is more closely associated with changes in dynamic lung function parameters than BMI (15). The weight-adjusted waist index (WWI), proposed by Park et al. (16); is more accurately reflects central obesity by capturing adipose tissue distribution. WWI combines the advantages of waist circumference, overcomes the limitations of BMI, and provides a more robust assessment of fat and muscle mass (17). This characteristic makes WWI potentially more reliable and sensitive in assessing obesity-related health risks.

PRISm represents a distinct pulmonary function phenotype potentially indicative of early COPD. Despite central obesity’s established role in respiratory pathophysiology, no studies have yet explored the relationship between WWI and PRISm. This study investigates (1) the association between WWI and PRISm, and (2) WWI’s linkage to all-cause mortality in U.S. adults with and without PRISm.

## 2 Methods

Publicly accessible data were derived from the National Health and Nutrition Examination Survey (NHANES) (18), which started releasing public-use data in 1999 and continues to do so every 2 years. To create a representative and randomized sample of the noninstitutionalized civilian population in the U.S., the NHANES data collection is designed with stratified, multistage, and cluster sampling (18). The study protocol was approved by the U.S. National Center for Health Statistics Institutional Review Board (Protocol #2005-06, #2011-17), and all participants provided written informed consent.

### 2.1 Study population

**Figure 1:**
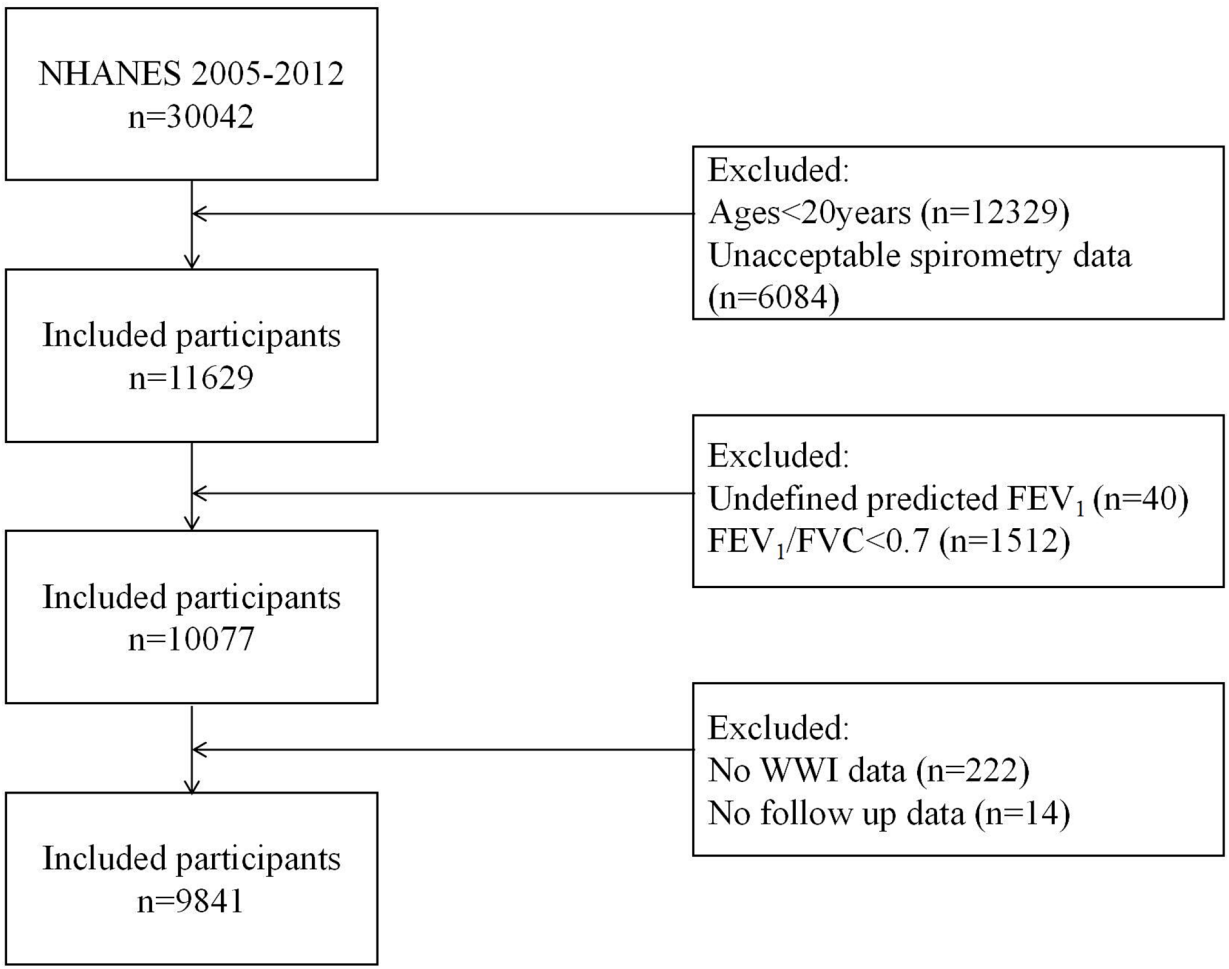
Study flow chart. NHANES, National Health and Nutrition Examination Survey; FEV_1_, forced expiratory volume in 1 second; FVC, forced vital capacity; WWI, weight-adjusted waist index.

We analyzed data from three NHANES cycles (2007–2012) based on spirometry availability. From 30,442 initial participants, 9,841 were included after applying exclusion criteria: (1) age <20 years (*n* = 12,329); (2) no spirometry examination or quality below grade B (*n* = 6,084); (3) airflow obstruction (FEV_1_/FVC <0.70; *n* = 1,512); (4) undefined predicted FEV_1_ (*n* = 40); and (5) missing WWI (*n* = 222) and follow-up data (*n* = 14) (Figure 1).

### 2.2 WWI assessment

WWI is an anthropometric statistic tool based on waist circumference and weight used to assess central obesity (16). WWI (cm/√kg) was calculated as waist circumference (cm) divided by the square root of weight (kg) (19). Waist circumference and weight were measured by professional health technicians using standardized protocols. In this study, WWI was utilized as an exposure variable, and participants were divided into Q1, Q2, Q3, and Q4 groups on the basis of the WWI quartiles for further analysis (20).

### 2.3 Lung function and PRISm definition

Spirometry data (FEV_1_, FVC) were extracted from NHANES SPX dataset. We only used datasets with quality grades A (highest quality, exceeding American Thoracic Society standards) and B (adequate technical quality and reproducibility). The detailed methods and quality assessments used for pulmonary function measurements have been described elsewhere (21). The predicted values of various lung function parameters were calculated via the NHANES III formula (22). PRISm was defined as FEV_1_/FVC of 0.70 or higher and FEV_1_ of less than 80% predicted (13, 23).

### 2.4 Ascertainment of survival outcome

The survival outcome in our study was all-cause mortality. The Centers for Disease Control and Prevention website provided data on deaths, which were linked to the NHANES database via unique subject identification (24). The death data were ascertained through December 31, 2019. The variables “MORTSTAT” and “PERMTH_EXM” were used to represent the death status and follow-up time, respectively.

### 2.5 Covariates

This study included a variety of characteristics as covariates to address potential confounding effects. Age, gender (male or female), race and ethnicity, educational attainment, marital status, the family poverty income ratio (PIR), smoking status, and physical activity were all self-reported via standardized questionnaires. BMI was predetermined in the downloaded NHANES dataset. Race and ethnicity were categorized into non-Hispanic White, non-Hispanic Black, Mexican-American, Hispanic other, and other. Educational attainment was delineated into three categories: under high school, completed high school, and above high school. Marital status was divided into two categories: married/living with a partner or widowed/divorced/separated/never married. Family income was further divided into three income groups on the basis of PIR: high-income earners (PIR ≥4), middle-income earners (PIR ≥1 and <4), and low-income earners (PIR <1) (23). On the basis of smoking history and current smoking status, smoking status was classified as never smoked (fewer than 100 cigarettes), former smoker (more than 100 cigarettes smoked in the past but not currently smoked), or current smoker (more than 100 cigarettes and frequently smoked) (25). Physical activity intensity was measured on the basis of participant reports of vigorous activity (high-intensity fitness, sports, and activity, such as basketball or running) and moderate physical activity (such as regular cycling, brisk walking, and swimming) (26).

### 2.6 Statistical analysis

All analyses performed in this study followed the criteria outlined in the Centers for Disease Control and Prevention guidelines and used the recommended weighting scheme (27). Continuous variables are presented as the mean ± standard deviation (SD) and were compared using the *t*-test or Wilcoxon rank-sum test, as applicable. Categorical variables are presented as counts (weighted proportions) and were compared via the Pearson chi-square test unless otherwise stated. Weighted univariate and multivariate logistic regression analyses were performed to estimate the association between WWI and PRISm, and the results are presented as odds ratios (ORs) with 95% confidence intervals (CIs). We conducted survival analysis across groups to investigate whether WWI is linked to the risk of all-cause mortality among individuals with or without PRISm. Kaplan–Meier (KM) survival curves were used to assess differences in survival probability over time by WWI quartiles, and between-group differences were tested using the log-rank test. Weighted univariate and multivariate Cox proportional hazards regression models were employed to explore the relationship between the WWI and all-cause mortality. The potential nonlinear or dose-response associations between the WWI and PRISm, as well as all-cause mortality were further assessed using restricted cubic spline (RCS) curves after adjusting for confounding factors. Three weighted logistic regression and Cox regression models were used to control for confounders: Model 1 (unadjusted); Model 2 (adjusted for age, sex, and race); and Model 3 (adjusted for age, sex, race, educational attainment, marital status, family PIR, BMI, smoking status, and physical activity). Covariates in our models have been carefully chosen on the basis of previous literature and factors that associated with lung function (23). All the statistical analyses were performed using R statistical software (version 4.3.2) with a significance threshold of *p* < 0.05 (two-sided).

## 3 Results

### 3.1 Participant characteristics

The baseline characteristics of the included participants, stratified by PRISm status, are described in Table 1. Among 9,841 participants, 864 (8.8%) were PRISm cases. The mean age was 46.4 ± 15.5 years, with 53.05% (*n* = 5,204) female. Weighted PRISm prevalence significantly differed by age, BMI, race, marital status, family PIR, smoking status, physical activity, WWI, and lung function parameters (all *p* < 0.05). Specifically, PRISm participants are more likely to be older, have a higher BMI and WWI, have lower family PIR, higher rates of former/current smoking, and lower vigorous physical activity levels.

**Table 1:** Baseline characteristics stratified by PRISm status (weighted %).

Characteristic	Total participants (*n* = 9,841)	PRISm (*n* = 864)	Non-PRISm (*n* = 8,977)	*p*
Age	44.4 (15.5)	49.5 (15.2)	43.9 (15.5)	**<0.001**
Gender, *n* (%)				0.331
Male	4,637 (46.97%)	393 (43.69%)	4,244 (47.17%)	
Female	5,204 (53.03%)	471 (56.31%)	4,733 (52.83%)	
BMI, kg/m^2^	29.2 (6.73)	31.8 (8.37)	29.0 (6.50)	**<0.001**
Race, *n* (%)				**<0.001**
Mexican American	1,706 (9.20%)	39 (2.52%)	1,667 (9.62%)	
Other Hispanic	1,118 (5.95%)	40 (2.93%)	1,078 (6.13%)	
Non-Hispanic White	4,088 (67.04%)	173 (38.40%)	3,915 (68.80%)	
Non-Hispanic Black	2,045 (10.85%)	516 (45.62%)	1,529 (8.71%)	
Other	884 (6.96%)	96 (10.53%)	788 (6.74%)	
Education, *n* (%)				0.093
Under high school	2,316 (15.66%)	207 (19.94%)	2,109 (15.39%)	
Completed high school	2,174 (21.40%)	214 (26.69%)	1,960 (21.07%)	
Above high school	5,345 (54.3%)	443 (53.37%)	4,902 (63.54%)	
Marital status, *n* (%)				**<0.001**
Married/Living with partner	3,899 (36.17%)	428 (46.42%)	3,471 (35.53%)	
Widowed/Divorced/Separated/Never married	5,934 (63.83%)	435 (53.58%)	5,499 (64.47%)	
Family PIR, *n* (%)				**0.003**
<1.0	1,874 (14.13%)	183 (19.85%)	1,691 (13.78%)	
≥1.0 and <4.0	4,604 (46.55%)	418 (50.12%)	4,186 (46.33%)	
≥4.0	2,566 (39.32%)	183 (30.03%)	2,383 (39.88%)	
Smoke status, *n* (%)				**0.002**
Never smoke	5,746 (58.91%)	464 (52.65%)	5,282 (59.29%)	
Former smoke	2,056 (21.71%)	183 (24.45%)	1,873 (21.63%)	
Current smoke	2,034 (19.39%)	217 (22.90%)	1,817 (19.08%)	
Physical activity, *n* (%)				**0.001**
Inactive	5,660 (54.84%)	534 (57.32%)	5,126 (54.69%)	
Moderate	2,249 (24.28%)	200 (26.61%)	2,049 (24.14%)	
Vigorous	434 (4.38%)	37 (4.34%)	397 (4.38%)	
Moderate and vigorous	1,498 (16.50%)	93 (11.74%)	1,405 (16.80%)	
WWI	10.9 (0.82)	11.1 (0.88)	10.9 (0.81)	**<0.001**
Lung function parameters				
FEV_1_, mL	3,166 (863)	2,226 (613)	3,256 (829)	**<0.001**
FVC, mL	3,939 (1,055)	2,859 (795)	4,043 (1,018)	**<0.001**
FEV_1_/FVC	0.80 (0.05)	0.78 (0.05)	0.81 (0.05)	**<0.001**

All values are presented as the mean ± SD or as counts (weighted proportions). PRISm, preserved ratio impaired spirometry; BMI, body mass index; PIR, poverty income ratio; WWI, weight-adjusted waist index; FVC, forced vital capacity; FEV_1_, forced expiratory volume in 1 second. Bold values indicate statistical significance (*p* < 0.05).

### 3.2 Associations between WWI and PRISm

**Figure 2:**
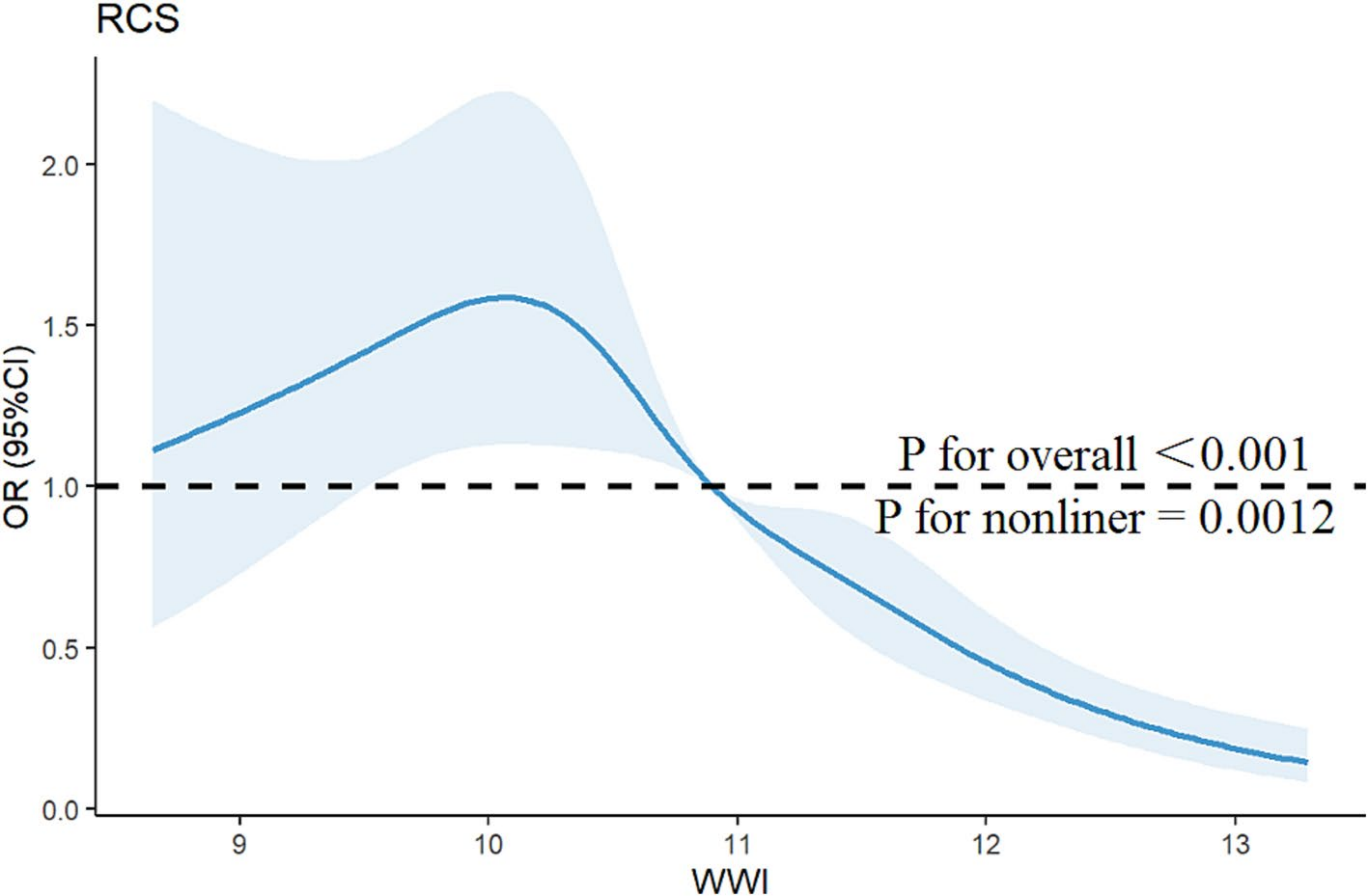
Restricted cubic spline curve for the association between the WWI and the risk of PRISm. The blue lines represent odds ratios, and the blue areas represent 95% confidence intervals. The model was adjusted for age, gender, race, education attainment, marital status, family PIR, BMI, smoking status, and physical activity.

The association between WWI and PRISm was investigated by using multivariate logistic regression analysis, as shown in Table 2. When WWI was analyzed as a continuous predictor, all the models revealed a negative association between WWI and the PRISm (all *p* < 0.05). For each unit increase in WWI, the risk of PRISm decreased by approximately 45% (OR = 0.55; 95% CIs: 0.47–0.65) after controlling for covariates. When WWI was converted from a continuous variable to a categorical variable (quartiles), the highest WWI quartile (Q4) showed significantly lower PRISm risk versus Q1 (reference), with Q3 demonstrating similar trends (non-significant in Q2). RCS analysis confirmed a nonlinear association (*p* for nonlinearity = 0.0012; Figure 2). When the WWI exceed 10.8974, the risk of PRISm decreased with increasing WWI. The results suggested that a lower WWI is associated with an increased risk of PRISm.

**Table 2:** ORs (95% *CIs*) for PRISm according to WWI, weighted.

WWI	Model 1			Model 2			Model 3		
	ORs	95% CIs	*p*	ORs	95% CIs	*p*	ORs	95% CIs	*p*
Continuous	0.59	0.52, 0.66	**<0.001**	0.53	0.46, 0.62	**<0.001**	0.55	0.47, 0.65	**<0.001**
Category									
Q1	1.0	1.0	1.0	1.0	1.0	1.0	1.0	1.0	1.0
Q2	0.90	0.70, 1.71	0.439	0.83	0.62, 1.11	0.213	0.85	0.63, 1.16	0.315
Q3	0.67	0.49, 0.90	**0.012**	0.61	0.44, 0.85	**0.006**	0.63	0.45, 0.88	**0.011**
Q4	0.36	0.29, 0.44	**<0.001**	0.32	0.24, 0.42	**<0.001**	0.33	0.25, 0.44	**<0.001**
*p* for trend			**<0.001**			**<0.001**			**<0.001**

Model 1: Unadjusted. Model 2: Adjusted for age, sex, and race. Model 3: Adjusted for age, sex, race, education attainment, marital status, family PIR, BMI, smoking status, and physical activity. ORs, odds ratios; CIs, confidence intervals; WWI, weight-adjusted waist index. Bold values indicate statistical significance (*p* < 0.05).

### 3.3 Correlation between WWI and all-cause mortality

**Figure 3:**
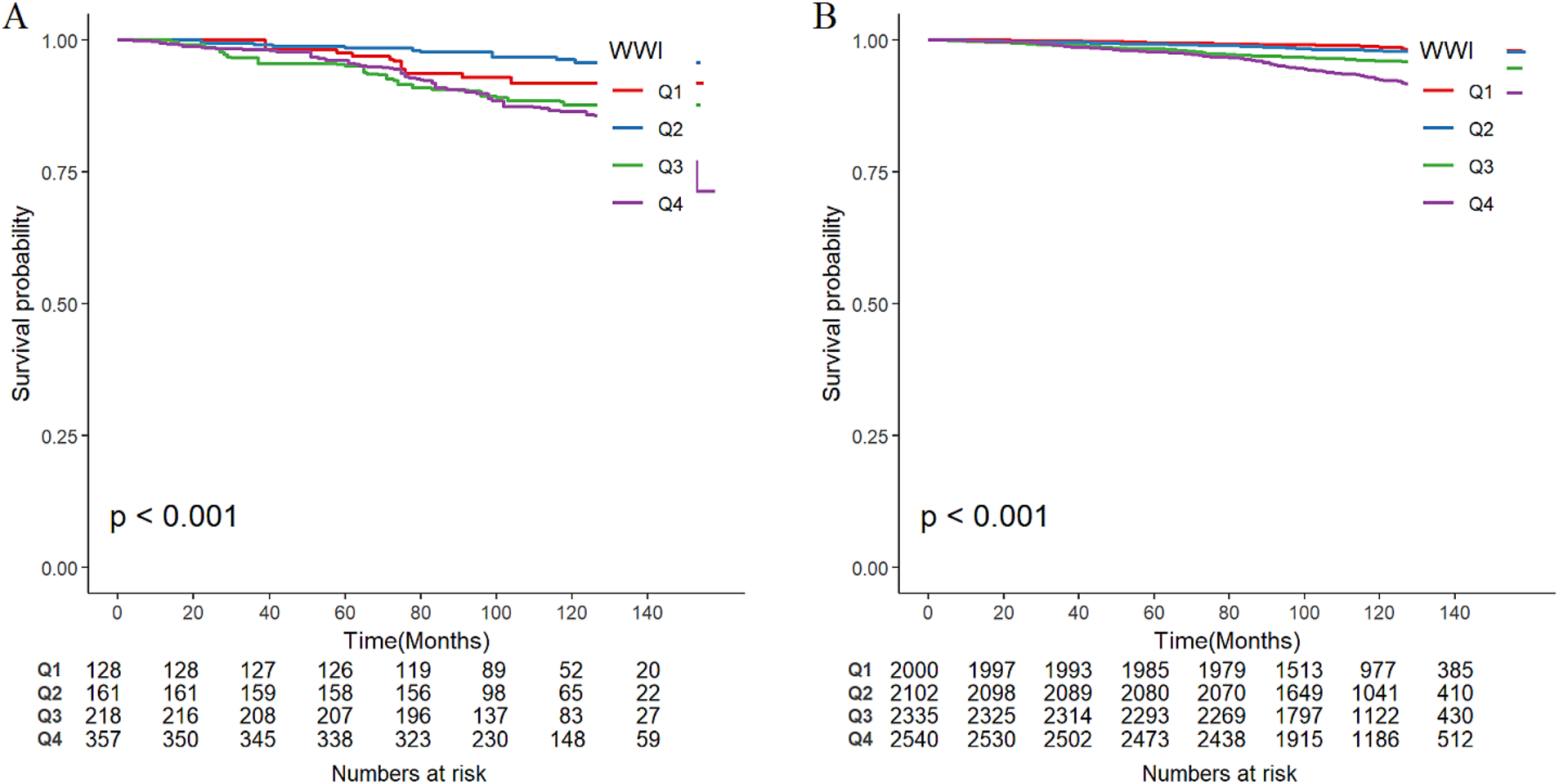
Weighted Kaplan–Meier survival curves of participants in different WWI groups and the incidence of all-cause mortality in **(A)** PRISm participants and **(B)** non-PRISm participants. Q1, Q2, Q3, and Q4 represent the quartile grouping of WWI values.

**Figure 4:**
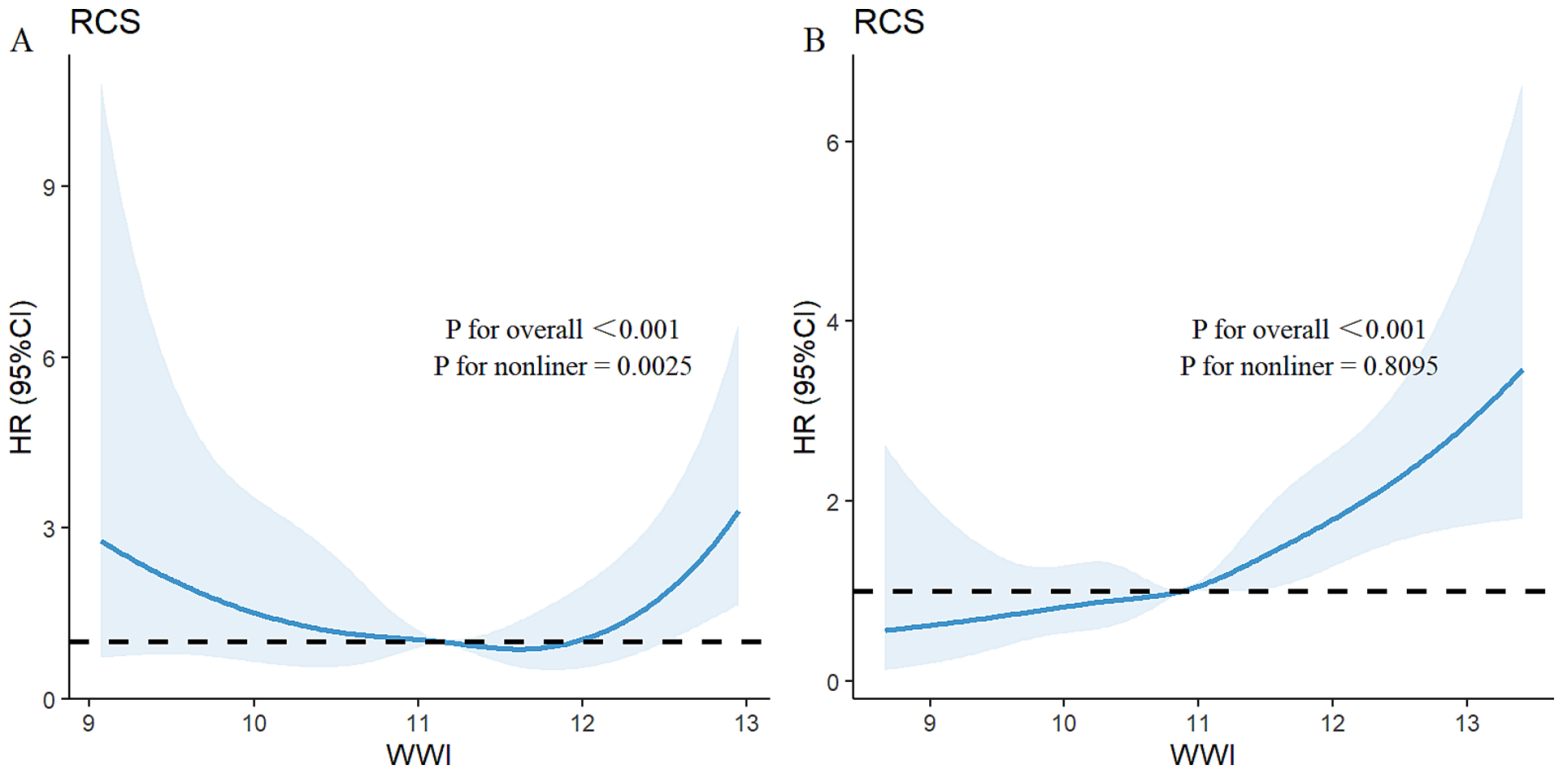
Adjusted restricted cubic spline curve for the association between the WWI and all-cause mortality in **(A)** PRISm participants and **(B)** non-PRISm participants. The blue lines represent references for hazard ratios, and the blue areas represent 95% confidence intervals. The model was adjusted for age, sex, race, education attainment, marital status, family PIR, BMI, smoking status, and physical activity.

Among the total participants, 543 individuals (5.52%) died from all causes. There were 115 deaths in the PRISm group, representing 13.31% of the PRISm population, compared with 428 deaths in the non-PRISm group, accounting for 4.77% of the non-PRISm population. KM curves revealed that all-cause mortality was greater in the Q4, Q3, and Q1 groups than in the Q2 group among the PRISm participants (all *p* < 0.05; Figure 3A). Both higher and lower WWI values were associated with an increased risk of mortality in PRISm population. Among non-PRISm participants, the risk of all-cause mortality was greater in the higher WWI group than in the lower WWI group (all *p* < 0.05; Figure 3B). Multivariate Cox regression models indicated that for each one-unit increase in the WWI, the adjusted hazard ratios (*HR*s) for mortality were 1.88 (95% CIs: 1.38–2.56) for the PRISm population and 2.32 (95% CIs: 2.07–2.59) for the non-PRISm population in Model 1 (Table 3). After adjusting for confounding factors, the risk of all-cause mortality in the Q2 group of PRISm participants was reduced. However, the associations of the other WWI groups with the risk of all-cause death in the PRISm population were not statistically significant. These results suggest a possible threshold effect between WWI and the risk of all-cause mortality in the PRISm population. The RCS analysis revealed that WWI was nonlinearly associated with the risk of all-cause mortality in the PRISm population (*p* for nonlinearity = 0.0025), and the curve was approximately U-shaped (Figure 4A). In contrast, there was a positive linear relationship with the non-PRISm population (*p* for nonlinearity = 0.8095) (Figure 4B).

**Table 3:** HRs (95% CIs) of PRISm status for all-cause mortality according to WWI, weighted.

WWI	Model 1			Model 2			Model 3		
	HRs	95% CIs	*p*	HRs	95% CIs	*p*	HRs	95% CIs	*p*
PRISm									
Continuous	1.88	1.38, 2.56	**<0.001**	1.40	0.87, 2.26	0.165	1.28	0.82, 1.99	0.282
Category									
Q1	Reference	Reference		Reference	Reference		Reference	Reference	
Q2	0.44	0.15, 1.29	0.135	0.25	0.18, 0.73	**0.012**	0.26	0.09, 0.71	**0.009**
Q3	1.52	0.71, 3.25	0.280	0.53	0.21, 1.33	0.178	0.45	0.18, 1.11	0.084
Q4	1.99	0.95, 4.18	**0.068**	0.58	0.21, 1.57	0.283	0.45	0.16, 1.26	0.128
*p* for trend			**0.003**			0.989			0.558
Non-PRISm									
Continuous	2.32	2.07, 2.59	**<0.001**	1.69	1.41, 2.03	**<0.001**	1.54	1.25, 1.91	**<0.001**
Category									
Q1	Reference	Reference		Reference	Reference		Reference	Reference	
Q2	1.39	0.82, 2.34	0.219	0.86	0.51, 1.45	0.567	0.88	0.51, 1.52	0.653
Q3	2.87	1.81, 4.57	**<0.001**	1.31	0.77, 2.21	0.320	1.24	0.71, 2.19	0.451
Q4	5.25	3.51, 7.85	**<0.001**	1.93	1.15, 3.25	**0.013**	1.74	0.96, 3.13	0.066
*p* for trend			**<0.001**			**<0.001**			**0.008**

Model 1: Unadjusted. Model 2: Adjusted for age, sex, and race. Model 3: Adjusted for age, sex, race, education attainment, marital status, family PIR, BMI, smoking status, and physical activity. HRs, hazard ratios; CIs, confidence intervals; WWI, weight-adjusted waist index. Bold values indicate statistical significance (*p* < 0.05).

We also analyzed the association between WWI and all-cause mortality in all participants. The results were similar to those of the non-PRISm population, which showed a positive linear relationship between WWI and all-cause mortality among all participants. The multivariate Cox regression results, KM curve, and RCS curve are provided in the Supplementary material.

## 4 Discussion

This study revealed the associations between WWI and PRISm as well as all-cause mortality using data from NHANES 2007–2012. We found a nonlinear relationship between WWI and PRISm, with lower WWI values associated with increased PRISm risk. Notably, WWI exhibited a U-shaped association with all-cause mortality in the PRISm population but a linear positive relationship in non-PRISm individuals and the overall cohort. Maintaining WWI within an optimal range may reduce mortality risk in PRISm patients.

Obesity is well known to be associated with a variety of diseases and an increased risk for mortality (28). However, the “obesity paradox” in COPD suggests that obesity/overweight may improve survival in COPD patients (29–31). Slight obesity is also considered a protective factor for lung function in people at risk of COPD (32). PRISm is a prodromal symptom of COPD, 22.2% to 35.8% of PRISm cases progress to COPD within 5 years (2, 8, 33). Recently, central obesity has been shown to be a significant risk factor for impaired lung function (34, 35). Research has proposed that FEV_1_ and FVC are better predicted by measurements of abdominal adiposity, such as waist circumference, than BMI (36, 37). In a 10-year cohort study, central obesity was found to be an independent risk predictor for COPD (38). In addition, another cohort study revealed that the visceral adiposity index (VAI) and lung function had a U-shaped association, with both excessively high and low VAI values being strongly linked to decreased lung function (39).

WWI is a novel obesity assessment index that plays a unique role in accurately reflecting central obesity (40, 41). Subsequent studies have also shown significant associations between WWI and visceral fat and muscle mass (42, 43). PRISm is a high-risk group of COPD patients, and there is still a lack of research on the management of PRISm. On the basis of previous studies related to obesity and COPD, we hypothesized that there might be some association between WWI and PRISm, and our study confirms this. The results revealed a nonlinear relationship between the WWI and PRISm, even after adjustment for confounders, including BMI. Fat distribution may be an important factor in the development of PRISm. Studies have shown that in the case of a high visceral fat load, the body may activate protective mechanisms or adjust metabolic pathways, such as antioxidant defense systems or anti-inflammatory molecules, to mitigate the effects of excessive fat accumulation in organs (44, 45). Some individuals with high WWI values may have greater subcutaneous fat storage capacity, which exerts a weaker effect on inflammation and metabolism than visceral fat and may even be protective (46, 47).

Given the older age, higher prevalence of obesity, and obesity-related comorbidities in PRISm (48), a higher all-cause mortality linked to PRISm than normal spirometry is not unexpected. Compared with normal spirometry, PRISm considerably increases the risks of respiratory disease, cardiovascular disease (CVD), and all-cause death (13, 49, 50). In real-world biomedical research, the effects of many research factors on outcome variables are not simple linear patterns but rather complex threshold effects. Specifically, these factors may have a positive or negative effect on the outcome variable within a certain range, but the direction of the effect changes once a certain cutoff point exceeds (51). For example, studies have reported a threshold effect between WWI and CVD risk (52). Another study revealed a nonlinear correlation between the WWI and all-cause mortality; only in the top quartile of WWI levels (≥11.2 cm/√kg) was the risk of mortality significantly increased (53). A similar phenomenon was found in our results. On one side, when WWI is greater than 10.8974, the effect of abdominal obesity on PRISm shifts from negative to positive. Apart from that, we found a U-shaped association between WWI and all-cause mortality in PRISm. Specifically, when the WWI was near the range of the second quartile, the risk of all-cause mortality was lowest in the PRISm population.

This phenomenon may be the result of a combination of various mechanisms. On the one hand, visceral fat is a metabolically active tissue that can release various proinflammatory cytokines, such as C-reactive protein (CRP), interleukin-6 (IL-6), and tumor necrosis factor-α (TNF-α) (54). Through systemic inflammatory responses, these proinflammatory substances impact lung tissue, resulting in oxidative stress and chronic inflammation (55). The infiltration of adipose tissue by activated macrophages and their interaction can also induce systemic inflammation, which may also contribute to pulmonary function impairment (56). In addition, fat deposition in the mediastinum and abdominal cavity alters breathing patterns and respiratory system compliance, which impair pulmonary function parameters through mechanical factors (57).

On the other hand, obesity may involve not only fat accumulation but also a decline in skeletal muscle mass. Research has demonstrated a correlation between sarcopenia and the severity of respiratory diseases (58). The WWI threshold effect may be linked to changes in muscle mass. Therefore, the impacts of muscle loss may offset the contribution of visceral fat to all-cause mortality in PRISm when the WWI surpasses a certain threshold. Second, obesity is a sign of lower cumulative tobacco exposure, and individuals who quit smoking gain more weight over time than do those who continue smoking (59). Among individuals with similar amounts of tobacco exposure, obesity might be protective against disease progression. The protective function of obesity in reducing mortality has been confirmed in COPD patients in another study, which revealed that a BMI greater than 30 kg/m^2^ was associated with increased spirometry parameters (FEV_1_ and FVC) (60). This dual effect of obesity and adipose tissue on the organism may account for the complex relationship between WWI and PRISm as well as all-cause mortality.

This is the first study to examine the association between central obesity and PRISm. We used an emerging obesity assessment metric, which offers new perspectives for future application of WWI in PRISm studies. However, several limitations of the study should be noted. First, the cross-sectional design of this study makes it impossible to determine a causal relationship between WWI and PRISm. Future prospective or longitudinal studies could be conducted to confirm causality, and Mendelian randomization could also be considered to strengthen causal inference. Second, because of the sample size limitations for postbronchodilator data, we used prebronchodilator spirometry values. Previous studies on PRISm utilizing NHANES data have been published with the same limitations (23, 61, 62). Future studies with larger sample sizes should use postbronchodilator data to more accurately assess the relationship between PRISm and WWI. Nevertheless, our findings still provide meaningful insights. Third, even though we adjusted for various potential confounders, such as demographic characteristics, lifestyle, and physical activity levels, we were unable to rule out all potential residual confounders, particularly unmeasured variables. Finally, because NHANES is limited to the U.S. adults, our results may lack representativeness for other countries and regions. Future studies should incorporate imaging data, inflammatory biomarkers (e.g., CRP, IL-6), or metabolic pathways (e.g., insulin resistance, oxidative stress) to investigate potential mechanisms through which WWI may influence PRISm. Clinical trials evaluating targeted interventions, such as central obesity control or weight management are warranted to assess their effects on PRISm progression and mortality.

## 5 Conclusion

The present study revealed that lower WWI value is associated with an increased risk of PRISm. We further identified a U-shaped association between WWI and all-cause mortality in the PRISm population, indicating both excessively low and high WWI levels elevate mortality risk. Central obesity likely mediates PRISm pathogenesis, and maintaining WWI within an optimal range may reduce mortality risk in individuals with PRISm. Integrating WWI assessment and targeted interventions into PRISm management protocols could mitigate disease progression and lower mortality rates.

## Data Availability

Publicly available datasets were analyzed in this study. This data can be found here: https://www.cdc.gov/nchs/nhanes/index.htm.
