# Supplementary material for: Association of weight-adjusted waist index with preserved ratio impaired spirometry and all-cause mortality
Source: Front Nutr. 2025 May 20;12:1594453. doi: 10.3389/fnut.2025.1594453 (PMC12129800; doi:10.3389/fnut.2025.1594453)
Supplement: Supplementary file 1 [file Data_Sheet_1.docx]

Supplementary Material

*Association of weight-adjusted waist index with preserved ratio impaired spirometry and all-cause mortality*

# Supplementary Figures and Tables

## Supplementary Figures


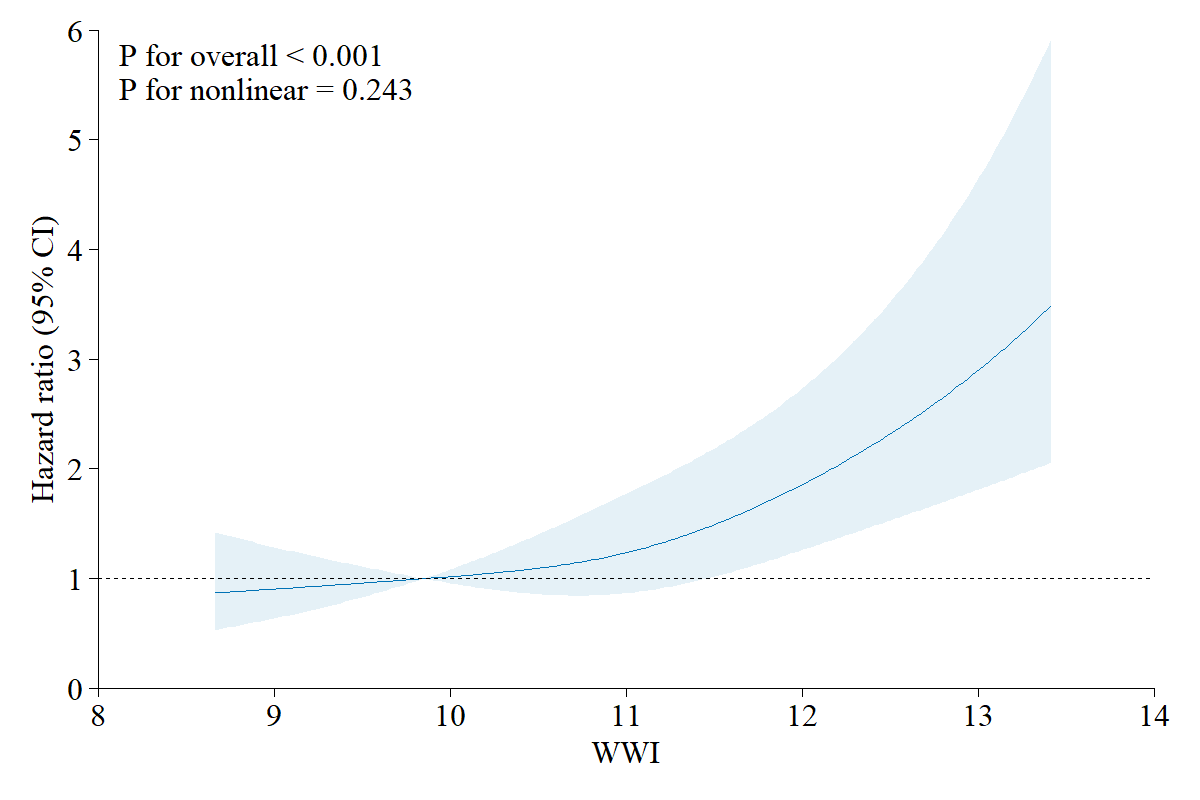


**Supplementary Figure 1.** Adjusted restricted cubic spline curve for the association between the WWI and all-cause mortality in general participants. The blue lines represent references for hazard ratios, and the blue areas represent 95% confidence interval. The model was adjusted for age, sex, race, education attainment, marital status, family PIR, BMI, smoking status, and physical activity.


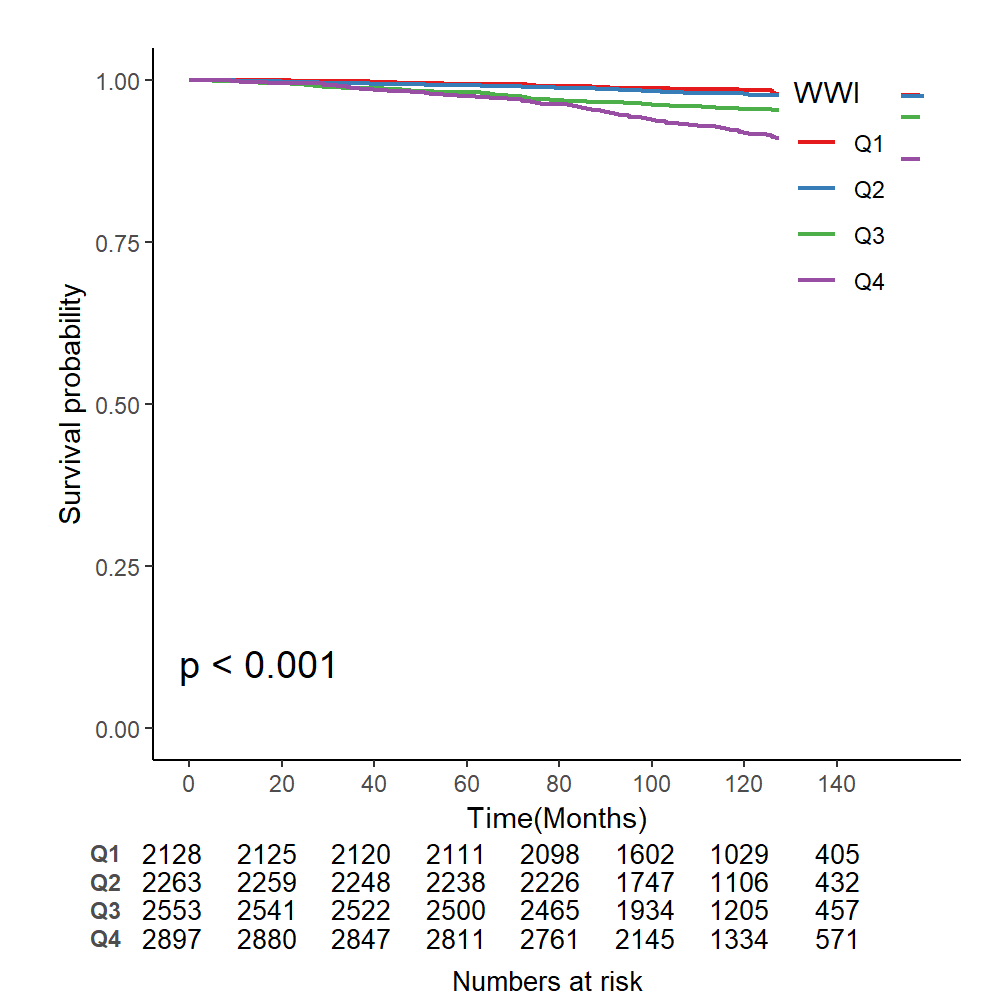


**Supplementary Figure 2.** Weighted Kaplan-Meier survival curves of participants in different WWI groups and the incidence of all-cause mortality in general participants. Q1, Q2, Q3, and Q4 represent the quartile grouping of WWI values.

## Supplementary Table

**TableS1** HRs (95% *CIs*) of all participants for all-cause mortality according to WWI, weighted

| **WWI** | **Model 1** | | | **Model 2** | | | **Model 3** | | |
| --- | --- | --- | --- | --- | --- | --- | --- | --- | --- |
|  | HRs | 95% *CIs* | *P* | HRs | 95% *CIs* | *P* | HRs | 95% *CIs* | *P* |
| Continuous | 2.36 | 2.09, 2.67 | **< 0.001** | 1.71 | 1.44,2.04 | **< 0.001** | 1.56 | 1.27, 1.96 | **< 0.001** |
| Category |  |  |  |  |  |  |  |  |  |
| Q1 | Reference | Reference |  | Reference | Reference |  | Reference | Reference |  |
| Q2 | 1.23 | 0.79, 1.91 | 0.358 | 0.76 | 0.49, 1.18 | 0.225 | 0.77 | 0.49, 1.22 | 0.267 |
| Q3 | 2.73 | 1.86, 3.99 | **< 0.001** | 1.22 | 0.79, 1.90 | 0.365 | 1.13 | 0.70, 1.83 | 0.612 |
| Q4 | 5.03 | 3.53, 7.15 | **< 0.001** | 1.80 | 1.15, 2.81 | **0.010** | 1.57 | 0.94, 2.64 | 0.088 |
| *P* for trend |  |  | **< 0.001** |  |  | **< 0.001** |  |  | **0.006** |

Model 1: Unadjusted. Model 2: Adjusted for age, gender, and race. Model 3: Adjusted for age, gender, race, education, marital status, family PIR, BMI, smoke status, and physical activity. HRs: hazard ratios, CIs: confidence intervals, WWI: Weight-adjusted waist index. Bold values indicate statistical significance (*P* < 0.05).
